# The association between common urogenital infections and cervical neoplasia – A nationwide cohort study of over four million women (2002–2018)

**DOI:** 10.1016/j.lanepe.2022.100378

**Published:** 2022-04-21

**Authors:** Filip Jansåker, Xinjun Li, Jenny Dahl Knudsen, Niels Frimodt-Møller, Christer Borgfeldt, Kristina Sundquist

**Affiliations:** a Center for Primary Health Care Research, Clinical Research Centre (CRC), Department of Clinical Sciences Malmö, Lund University, Skåne University Hospital, Jan Waldenströms gata 35, Malmö 205 02, Sweden; b Department of Clinical Microbiology, Rigshospitalet, Copenhagen, Denmark; c Department of Obstetrics and Gynaecology, Department of Clinical Sciences Lund, Skåne University Hospital, Lund University, Sweden; d Center for Community-based Healthcare Research and Education (CoHRE), Department of Functional Pathology, School of Medicine, Shimane University, Japan; e Department of Family Medicine and Community Health, Department of Population Health Science and Policy, Icahn School of Medicine at Mount Sinai, NY, USA

**Keywords:** Bacterial vaginosis, Cervical cancer, Cystitis, Sociodemographic factors, Urogenital infections, Vaginal infections

## Abstract

**Background:**

Cervical cancer is a major cause of mortality and morbidity in women worldwide. This study aimed to estimate the association between common urogenital infections and cervical neoplasia.

**Methods:**

A multi-register national cohort study of 4,120,557 women aged ≥15 years (2002–2018) was conducted. The outcomes were cervical cancer and carcinoma *in situ* (Swedish Cancer Register). The main predictors were urogenital infections—(urinary) cystitis, (bacterial) vaginosis, (candida) vulvovaginitis. Incidence rates per 10,000 person-years were calculated (using the European Standard Population). Cox regression was used to estimate hazard ratios (HR) while adjusting for possible confounders—other genital infections (e.g., cervicitis, salpingitis, urogenital herpes), parity, and sociodemographic factors.

**Findings:**

In 39·0 million person-years of follow-up, the incidence rate for cervical cancer was 1·2 (95% CI 1·1–1·2) per 10,000 person-years and the figure for cervical carcinoma *in situ* was more than tenfold higher. The fully adjusted HRs for cervical cancer were 1·31 (95% CI 1·15 and 1·48) and 1·22 (95% CI 1·16 and 1·29) for vaginosis and cystitis, respectively. Vaginosis showed a gradient association to carcinoma *in situ.* Vulvovaginitis was inversely associated with cervical cancer, but not significantly related with carcinoma *in situ* in the fully adjusted model. A temporal association with cervical cancer was observed for vaginosis and vulvovaginitis (inversely) but not for cystitis.

**Interpretation:**

In this large nationwide cohort of women, medically attended common urogenital infections were independently associated with cervical neoplasia, but cystitis was not temporally associated with cervical neoplasia. These findings could be used to increase focus on preventive measures, HPV-vaccination programmes, HPV-analyses- and cervical cancer screening, especially in women suffering from vaginosis. Future studies on the causal mechanism are warranted before generalized public health recommendations can be made.

**Funding:**

Region Skåne, Tore Nilsons Stiftelse, and Swedish Society of Medicine.

**Research in context**

**Evidence before this study**

Cervical cancer is a major cause of mortality and morbidity in young adult and older women. It develops in almost all patients from a carcinogenic human-papillomavirus (HPV) cervical infection, which causes cervical carcinoma *in situ* that can progress to cancer. Several risk factors of HPV infections are shared with common urogenital infective conditions, such as cystitis (lower urinary tract infection), vaginosis, and vulvovaginitis, e.g., sexual behaviour and certain vaginal microbiome structures. Sexually transmitted infections such as urogenital herpes and chlamydia have been found to facilitate the development of cervical neoplasia from HPV infections. The relationship with more common urogenital infective conditions remains less clear. Previous small studies of heterogenous designs have suggested bacterial vaginosis to be an independent risk factor for cervical neoplasia, while vulvovaginitis has been inversely associated with this. However, to the best of our knowledge, no large population-based study has explored this on comprehensive sets of healthcare data including known confounders. Furthermore, we were unable to locate any large study that had investigated the relationship between cystitis and cervical neoplasia. This is likely due to the previous lack of large population-based primary healthcare data, which is where most urogenital infections are diagnosed and managed.

**Added value of this study**

Using nationwide primary healthcare data linked with the national cancer register and other registers in Sweden, this study identified bacterial vaginosis and cystitis as being independently associated with cervical cancer, adjusted for known confounders. It also shows vulvovaginitis to be inversely associated with cervical cancer. Bacterial vaginosis and cystitis were also associated with cervical carcinoma *in situ*, but vulvovaginitis was not, in the fully adjusted model. The risk for cervical carcinoma *in situ* also seems to be amplified with an increased number of bacterial vaginosis and cystitis events. Temporal associations to cervical cancer were seen for bacterial vaginosis and vulvovaginitis (inversely) but not for cystitis.

**Implications of all the available evidence**

Although HPV-vaccination is an important intervention for breaking the causal mechanism in cervical cancer development, screening programs remain critical in the prevention of cervical cancer worldwide. The findings in this present study can be used to identify women at higher risk of developing cervical cancer. Women with high morbidity of urogenital infections might be offered preventive interventions such as HPV-screening, especially if the woman is not actively participating in the cervical cancer screening program and/or without HPV-vaccination. In particular, women suffering from bacterial vaginosis might, already at the same time as diagnosis, benefit from such offers and interventions. However, more studies confirming our findings and on causal mechanisms are needed before any generalized healthcare recommendations can be made.

Alt-text: Unlabelled box

## Introduction

Urogenital infections such as bacterial vaginosis, vulvovaginal candidiasis (vulvovaginitis), and acute lower urinary tract infections (cystitis) cause significant annual morbidity in women worldwide with regional and socioeconomic variabilities and where the conditions seem to share several risk factors such as vaginal dysbiosis, sociodemographic factors, and certain sexual behaviours.^1^, ^2^, ^3^, ^4^, ^5^, ^6^, ^7^ In nationwide data, we recently found that almost every third adult women have had at least one episode of cystitis during a two decade period,^6^ compared to about one in twenty in regard to bacterial vaginosis and vulvovaginitis.^7^ Both these studies demonstrated considerable sociodemographic variability related to the distribution of these common urogenital infections.

Depletion of certain possible commensal bacteria seem to facilitate augmentation of certain pathogens in the vagina and has been associated with urogenital infections as well as oncogenic cervical infections and neoplasia.^4^^,^^5^^,^^8^, ^9^, ^10^, ^11^, ^12^, ^13^, ^14^, ^15^, ^16^, ^17^, ^18^ Therefore, it is possible that these common urogenital infections could be related to cervical cancer, which is mainly caused by persistent human-papilloma-virus (HPV) infection.^19^, ^20^, ^21^ The evidence also suggests that sexually transmitted genital infections seem to act as co-factors for HPV^21^^,^^22^ infection in the development of cervical neoplasia.^20^^,^^21^ The relationship of other and more common urogenital infections to cervical cancer has, however, not been as well examined.

Previous studies have found correlations between bacterial vaginosis and cervical neoplasia,^23^^,^^24^ but on the other hand, vaginal microecological conditions, such as vulvovaginal candidiasis, have been suggested to be inversely associated with cervical neoplasia.^24^ However, these studies have been mostly cross-sectional and heterogeneous in design.^23^^,^^24^ Thus, large scale studies are needed to fully establish any relationship between common urogenital infections and cervical cancer on a population-based level, which could help improve the preventive work on this common malignancy in women. To the best of our knowledge, no nationwide study exists on this matter, especially not for cystitis. This is likely due to the previous lack of nationwide primary healthcare data where cystitis and common vaginal conditions such as bacterial vaginosis, and vulvovaginitis usually occur.

The hitherto scarce availability of population-based data has also made it difficult to conduct large scale studies exploring urogenital risk factors for cervical neoplasia and adjusted for potential confounders. The evidence suggests that several possible factors might be associated with urogenital infections as well as cervical cancer, such as age and other sociodemographic factors, parity, sexual behaviour, and sexually transmitted infections.^2^, ^3^, ^4^^,^^14^^,^^20^, ^21^, ^22^, ^23^, ^24^, ^25^, ^26^ Similar to urogenital infections,^6^^,^^7^ sociodemographic differences have also been described related to cervical cancer screening in Sweden.^27^

With this study, we aim to explore the possible relationship between common medically attended urogenital infections and cervical cancer, adjusted for sociodemographic factors, parity, and other genital infections.

## Methods

### Study design, population, and setting

A nationwide open cohort study was conducted on women ≥15 years during the period 2002 to 2018 in Sweden. Baseline occurred when a woman residing in Sweden was ≥15 years or immigrated to Sweden and was ≥15 years of age somewhere during the study period. The STROBE statement-checklist for cohort studies was considered when conducting the study and writing the manuscript. The research was conducted at Lund University, Sweden.

### Ascertainment of the outcome variables (cervical neoplasia)

The outcomes were identified in the nationwide Swedish Cancer Register during the study period. The study used the 7^th^ revision of the International Classification of Diseases (ICD-7) codes used in the Swedish Cancer Register. The first analysis was conducted on (invasive) *cervical cancer* as outcome. This was measured as ICD-7 code 171 histologically labelled as tumour indication of malignant neoplasia of cervix uteri. The second analysis was conducted on non-invasive (*in situ*) neoplasia of cervix uteri (in this paper: *cervical carcinoma in situ);* measured as the ICD-7 code 171 histologically labelled as tumour indication of benign neoplasia of cervix uteri. Each woman could only be included once for each outcome during the study period. About 16,100 participants with a registration of ICD-7 code 171 in the Swedish Cancer Register prior to the start of the study period were excluded from the study population (0·4%). In a sensitivity analysis all women with an outcome diagnosis prior to the exposure (urogenital infection) were excluded (Table 5).

### Ascertainment of predictor variables (urogenital infections)

The clinical ICD-10 diagnoses of B373 (“Candidiasis of vulva and vagina”), N30 (“Cystitis”) and N768 (“Other specified inflammation of vagina and vulva”) were used to measure three common medically attended urogenital infective conditions: candida vaginitis (referred to as *vulvovaginitis*), acute infective cystitis (referred to as *cystitis*), and bacterial vaginosis. The predictors were measured categorically and continuously (number of infections) as diagnoses using nationwide primary health care data and the National Outpatient Register during the study period. The codes were used in primary healthcare in Sweden during the study period.^6^^,^^7^ The ICD-10 codes N301-4 and N308 were not considered as these were not in-line with acute infective lower urinary tract infection (cystitis). Bacterial vaginosis does not have a specific ICD-10 code in Sweden, but N768 is most often used for this condition, and is the only code that is mentioned when using one of the most used clinical practice online tools in Sweden (in Swedish: www.internetmedicin.se/behandlingsoversikter/gynekologi-obstetrik/bakteriell-vaginos/). The ICD-10 diagnostic code N768 was thus used as a proxy for the medically attended condition of bacterial vaginosis. Each woman was a unique participant and could only be included once. The three types of urogenital infections were identified as the first infection diagnosed in primary healthcare settings and out-patient specialist care. Analyses were done for each predictor separately. The number of infections were also identified during the study period. Women with more than one type of urogenital infection were also measured (Supplementary Table S1). An additional analysis was done which included all urogenital infections in one combined analysis.”

### Ascertainment of potential confounders

Considering the possibilities for potential confounders, sociodemographic factors, parity, and other genital infections of suspected sexually transmitted geneses were included in the analyses. *Sociodemographic factors* were measured as: *Age groups* defined as being between 15 and 24, 25 and 34, 35 and 44, 45 and 64, or ≥65 years of age; *Family income quartiles; Individual education level* was measured as high school education or less (≤12 years) or at least one year of university or college education (>12 years). Highest *family education level* was used if the participant was <18 years of age at baseline; *Region of residence* was categorized into three groups (i.e. residing in large cities, or outside of large cites in Southern- or Northern Sweden; *Country of origin* was defined as originating from any of the following countries/regions: Sweden (born in); Eastern Europe; Western countries; Middle East/North Africa (MENA); Africa (excluding North Africa); Asia (excluding Middle East) and Oceania; or Latin America and the Caribbean. *Parity* was measured as nullipara or unipara and above. The definitions used for these variables were similar to previous studies by our group.^6^ *Other genital infections* were measured as ICD-10 codes A600 (urogenital herpes simplex) and N70-5 (Bartholin gland infection, cervicitis, pelvic inflammatory disease, uteritis, salpingitis etc). *Marital status* was also considered in a sensitivity analysis, measured as married/cohabiting or not.

### Data sources

The register used to identify the outcomes was the nationwide Swedish Cancer Register. Nationwide Swedish primary healthcare data and the National Patient Register (outpatient data) were used to identify the main predictors and medical confounders. The coverage of the nationwide primary healthcare data used varied over time and region. Today, it includes primary healthcare data from 95% of the Swedish administrative regions. The time-periods differed depending on the time of digitalisation of the patient records in a certain region and >90% of the population was included in this study.^6^ The Total Population Register, which was used to collect data on the sociodemographic variables and emigration, was nearly 100% complete for the entire national population. Other registers used in this study were the National Patient Register, which includes hospital diagnoses, the Cause of Death Register, and the Swedish Medical Birth Register. All linkages between the registry data were performed using a pseudonymised version of the unique 10-digit personal identification number assigned to each person for their lifetime upon birth or immigration to Sweden. The analyses did not exclude observations with missing values. For age and country of origin <0·0% values were missing. Values missing for education (2·5%), family income (3·5%), and region of residency (2·0%) were included in the group with the lowest level of education, lowest level of family income, and living in large cities. Data of the study population can be viewed in Supplementary Material (Table S5).

### Statistical analysis

Descriptive statistics on the study population, total person-years of follow-up, number of first events of the outcomes, and incidence rates per 10,000 person-years were calculated for each predictor variable and standardised for age based on the European Standard Population.^28^ To assess the association between the predictor variables and the outcomes, Cox regression models were used to estimate hazard ratios (HR) and 95% confidence intervals (CI). The study period started on January 1, 2002, and person‐years were calculated from the age of 15 years until an outcome event, death, emigration, or end of the study period (December 31, 2018). Four models were used for each of the three main predictors (analysed separately): Model 1, a crude model; Model 2, age adjusted; Model 3 adjusted for all sociodemographic factors; and Model 4, adjusted for all potential confounders. All three predictors were also analysed together in a fully adjusted model. The number of events associated with the outcomes were also analysed for each predictor adjusted for all possible confounders, including linear trend estimations. We also conducted a sensitivity analysis taking the time of infection in relation to the outcomes into account. In this model we excluded women diagnosed with cervical cancers and cervical carcinoma *in situ* prior to the urogenital infection (exposure). To examine that the strength of the associations did not change over time, proportionality assumptions were checked by plotting the incidence rates over time and by calculating Schoenfeld (partial) residuals – these assumptions were fulfilled. Kaplan-Meier curves were also plotted (Supplementary Material). Potential interactions between urogenital infections and country of origin were examined in relation to cervical cancer – no significant interactions were found. A two-tailed *p*-value of <0·05 was used to determine statistical significance. SAS software version 9.4 (SAS Institute Inc.; Cary, NC, USA) was used for all statistical analyses.

### Ethical consideration

The present study was a non-intervention nationwide register study of pseudonymised secondary data obtained from national authorities and approved by the Ethical Review Board in Lund, Sweden. All methods were performed in accordance with the relevant guidelines and regulations.

### Role of funding source

The funding sources of the study were all non-commercial and had no role in the study design; the collection, analysis, and interpretation of data; the writing of the report; or in the decision to submit the paper for publication.

## Results

The study population consisted of 4,120,557 women with a total follow-up of around 39·0-million person-years. Cystitis was the most common urogenital infection in the study population, accounting for 13·2-million person-years, considerably more than bacterial vaginosis (1·4-million person-years) and vulvovaginitis (1·9-million person-years). About half of the women who had been diagnosed with bacterial vaginosis or vulvovaginitis had suffered at least one other urogenital condition as well during the study period (Supplementary Table S1).

Table 1 shows that the incidence rate of cervical cancer was 1·2 (95% CI 1·1 to 1·2) per 10,000 person-years with a binomial age distribution with lowest incidence rates in the youngest women and women between 45 and 64 years of age at baseline. Women with a history of bacterial vaginosis followed by cystitis had higher incidence rates than the general population, while women with a history of vulvovaginitis had a lower incidence rate. The incidence rates of cervical carcinoma *in situ* were in general over tenfold higher than cervical cancer. Women with a history of urogenital infections had higher incidence rates than those without. The incidence rate for cervical carcinoma *in situ* was highest in the youngest age group and decreased gradually by increases in age.

**Table 1:** Study population, number of cases and incidence rate (per 10,000 person-years) of invasive cervical cancer and cervical carcinoma *in situ* (2002–2018).

			Cervical cancer					Cervical carcinoma *in situ*				
	Total population		Number of unique events		Incidence rate per 10,000 person-years			Number of unique events		Incidence rate per 10,000 person-years		
	No.	%	No.	%	IR*	95% CI.		No	%	IR*	95% CI	
**Urogenital conditions**												
Cystitis	1,194,561	29·0	2209	38·2	1·3	1·3	1·4	22,778	36·6	14·6	14·4	14·8
Vaginosis	124,287	3·0	277	4·8	1·4	1·2	1·5	6412	10·3	23·8	23·2	24·4
Vulvovaginitis	167,864	4·1	221	3·8	0·9	0·8	1·0	5194	8·3	14·9	14·5	15·3
**Age at baseline** (years)												
15–24	674,120	16·4	634	11·0	1·0	0·9	1·1	26,301	42·3	41·4	40·9	41·9
25–34	677,650	16·4	1239	21·4	1·9	1·8	2·0	21,196	34·1	32·8	32·3	33·2
35–44	635,122	15·4	1100	19·0	1·7	1·6	1·8	9613	15·4	14·7	14·5	15·0
45–64	1,174,811	28·5	1588	27·5	1·3	1·2	1·4	4657	7·5	3·8	3·7	3·9
≥ 65	958,854	23·3	1220	21·1	1·6	1·5	1·7	482	0·8	0·6	0·6	0·7
**Total population**	4,120,557		5781		1·2	1·1	1·2	62,249		13·4	13·3	13·5

⁎ IR: Incidence rate per 10,000 person years, standardised based on the European Standard Population; CI: Confidence interval. Cystitis: Acute lower urinary tract infection. Vaginosis: Bacterial vaginosis. Vulvovaginitis: Candidiasis of vulva and vagina. Other genital infections: Bartholin gland infection, cervicitis, pelvic-inflammatory-disease, salpingitis, urogenital herpes, and uteritis.

Table 2 shows the association between the three urogenital infections and cervical cancer and cervical carcinoma *in situ*. Compared to no history of the corresponding urogenital infection, the HRs for cervical cancer were higher with bacterial vaginosis and cystitis but lower with vulvovaginitis both in the crude as well as the adjusted models. Compared to no history of the corresponding urogenital infection, the HRs for cervical carcinoma *in situ* were higher with bacterial vaginosis, cystitis and vulvovaginitis both in the crude as well as the adjusted models. Kaplan-Meier survival estimates on the urogenital infections and cervical cancer and cervical carcinoma *in situ* are shown in the supplementary material (Figures S1 and S2).

**Table 2:** The association between three common urogenital infections (analysed separately) and cervical cancer and cervical carcinoma *in situ*.

	No. Cervical neoplasia	Person- years	Model 1				Model 2				Model 3				Model 4			
Covariates			HR	95% CI		*P*-value	HR	95% CI		*P*-value	HR	95% CI		*P*-value	HR	95% CI		*P*-value
	**Cervical cancer (5781 events)**																	
Cystitis																		
(ref. none)	2209	13,248,433	1·23	1·17	1·30	<·0001	1·25	1·18	1·32	<·0001	1·25	1·18	1·32	<·0001	1·22	1·16	1·29	<·0001
Vaginosis																		
(ref. none)	277	1,383,417	1·39	1·23	1·57	<·0001	1·42	1·25	1·60	<·0001	1·42	1·26	1·60	<·0001	1·30	1·15	1·47	<·0001
Vulvovaginitis																		
(ref. none)	221	1,882,046	0·80	0·70	0·91	0·0008	0·80	0·70	0·92	0·0014	0·84	0·74	0·97	0·0133	0·80	0·70	0·91	0·0011
	**Cervical carcinoma *in situ* (62,249 events)**																	
Cystitis																		
(ref. none)	22,778	13,248,433	1·12	1·10	1·14	<·0001	1·11	1·09	1·13	<·0001	1·13	1·11	1·15	<·0001	1·10	1·08	1·12	<·0001
Vaginosis																		
(ref. none)	6421	1,383,417	3·12	3·04	3·20	<·0001	1·86	1·82	1·91	<·0001	1·91	1·86	1·96	<·0001	1·80	1·75	1·85	<·0001
Vulvovaginitis																		
(ref. none)	5194	1,882,046	1·79	1·74	1·84	<·0001	1·11	1·08	1·14	<·0001	1·18	1·15	1·21	<·0001	1·13	1·10	1·16	<·0001

HR: Hazards ratio. CI: Confidence interval. Cystitis: Acute lower urinary tract infection. Vaginosis: Bacterial vaginosis. Vulvovaginitis: Candidiasis of vulva and vagina. Model 1: Crude model; Model 2: Age adjusted model; Model 3: Adjusted for sociodemographic factors (age, education level, income quartile, region of residency, and country of origin), and parity. Model 4: Fully adjusted for sociodemographic factor, parity, and other genital infections (Bartholin gland infection, cervicitis, pelvic-inflammatory-disease, salpingitis, urogenital herpes, and uteritis).

Table 3 includes all urogenital infections and other genital infections in the adjustments. Around 3·5% (145,168 cases) of the population had suffered at least one event of other genital infections during the study period. This proportion was higher in women with cervical cancer 7·4% (incidence rate: 2·5 [CI 95 %; 2·3 to 2·8]) and cervical carcinoma *in situ* 10·2% (incidence rate: 21·3 [CI 95 %; 20·7 to 21·8]). The fully adjusted HRs for cervical cancer and cervical carcinoma *in situ* were significantly associated with other genital infections and remained significantly associated with cystitis and bacterial vaginosis. The inverse association between vulvovaginitis and cervical cancer also persisted, but no association with vulvovaginitis and cervical carcinoma *in situ* was found. When including marital status in the adjustments the results remained more or less unchanged and statistical significance was found in the association between vulvovaginitis and carcinoma *in situ* (Table S2).

**Table 3:** The fully adjusted association between three common urogenital infections (analysed together) and cervical cancer and carcinoma *in situ*.

	Cervical cancer						Cervical carcinoma *in situ*					
Covariates	No. events	Person-years	HR*	95% CI		*P*-value	No. events	Person-years	HR*	95% CI		*P*-value
**Cystitis**												
(ref. none)	2209	13,248,433	1·22	1·16	1·29	<·0001	22,778	13,248,433	1·07	1·05	1·09	<·0001
**Vaginosis**												
(ref. none)	277	1,383,417	1·31	1·15	1·48	<·0001	6421	1,383,417	1·77	1·73	1·82	<·0001
**Vulvovaginitis**												
(ref. none)	221	1,882,046	0·75	0·65	0·86	<·0001	5194	1,882,046	1·03	1·00	1·06	0·0734
**Other genital infections**												
(ref. none)	429	1,587,309	1·87	1·69	2·07	<·0001	6359	1,587,309	1·50	1·46	1·54	<·0001

HR: Hazards ratio. CI: Confidence interval. Cystitis: Acute lower urinary tract infection. Vaginosis: Bacterial vaginosis. Vulvovaginitis: Candidiasis of vulva and vagina. Other genital infections: Bartholin gland infection, cervicitis, pelvic-inflammatory-disease, salpingitis, urogenital herpes, and uteritis. *Fully adjusted for sociodemographic factor (age, education level, income quartile, region of residency, and country of origin), and parity.

Table 4 (data from Table S3) shows that the fully adjusted HRs associated with carcinoma *in situ* gradually increased with increased number of bacterial vaginosis events (linear trend *P*-value <·001). No clear gradient was found for cystitis, but the linear trend was statistically significant (*P*-value <·001). No gradient between the number of bacterial vaginosis events and cervical cancer was identified although five or more events of bacterial vaginosis were associated with the highest HR. We found no gradient between the number of cystitis- or vulvovaginitis events and cervical cancer, nor between vulvovaginitis and carcinoma *in situ* (Table S3). Furthermore, women with very high numbers of bacterial vaginosis or cystitis (i.e., six to ten or above ten events) seemed to be suffering an even higher risk of cervical carcinoma *in situ* during the study period compared to women with no registered infections (Table S4). However, statistical significance was absent in women with more than ten cystitis events.

**Table 4:** The association between number of urogenital infections and cervical cancer and carcinoma *in situ*.

	Bacterial vaginosis												Cystitis					
	Cervical cancer						Cervical carcinoma *in situ*						Cervical carcinoma *in situ*					
Covariates	No.	Person years	HR*	95% CI		*P*-value	No	Person years	HR*	95% CI		*P*-value	No.	Person years	HR*	95% CI		*P*-value
**Number of infections**^§^ (ref. Non)																		
One	189	927,849	1.33	1·15	1·54	0·0001	4086	927,849	1·73	1·68	1·79	<·0001	10,627	5,881,414	1·10	1·07	1·12	<·0001
Two	58	296,864	1·26	0·97	1·63	0·0852	1417	296,864	1·84	1·75	1·94	<·0001	4976	2,740,039	1·11	1·08	1·15	<·0001
Three	15	76,288	1·23	0·74	2·05	0·4179	416	76,288	1·96	1·78	2·16	<·0001	2598	1,481,920	1·11	1·07	1·16	<·0001
Four	5	41,739	0·75	0·31	1·80	0·5137	230	41,739	2·04	1·79	2·32	<·0001	1520	90,0178	1·12	1·06	1·18	<·0001
Five or more	10	40,677	1·50	0·81	2·79	0·2026	263	40,677	2·44	2·16	2·75	<·0001	3057	2,244,882	1·09	1·05	1·13	<·0001
			*Linear trend P-value = 0·7418*						*Linear trend P-value <·001*						*Linear trend P-value <·001*			

Data from Supplementary Table S3. HR: Hazards ratio. CI: Confidence interval. No.: Number of events. *Fully adjusted for sociodemographic factor (age, education level, income quartile, region of residency, and country of origin), parity, and other genital infections (Bartholin gland infection, cervicitis, pelvic-inflammatory-disease, salpingitis, urogenital herpes, and uteritis). § During the time period: 2002–2018.

In the sensitivity analysis (Table 5), in which women with cervical cancer and cervical carcinoma *in situ* prior to the urogenital infection were excluded, bacterial vaginosis remained significantly associated with both cervical cancer and carcinoma *in situ.* Vulvovaginitis remained inversely associated with cervical cancer and seemed to be slightly so with cervical carcinoma *in situ* as well: i.e., HR of 0·97 (95% CI 0·94–1·00; *P*-value = 0·0519). No association between cystitis and cervical cancer or cervical carcinoma *in situ* was observed in this model.

**Table 5:** The fully adjusted sensitivity analysis on the association between three common urogenital infections (analysed together) and cervical cancer and carcinoma *in situ,* after exclusion of cervical cancer and carcinoma *in situ* diagnosed within three years prior to the urogenital infection (exposure).

	Cervical cancer				Cervical carcinoma *in situ*			
**Exposure**	HR	95% CI		*P*-value	HR	95% CI		*P*-value
**Cystitis**^a^	1·03	0·97	1·09	0·3217	1·00	0·99	1·02	0·8586
(ref. non)								
**Vaginosis**^b^	1·16	1·01	1·32	0·0305	1·56	1·52	1·61	<·0001
(ref. non)								
**Vulvovaginitis**^c^	0·63	0·54	0·74	<·0001	0·97	0·94	1·00	0·0519
(ref. non)								

HR: Hazards ratio. CI: Confidence interval. Cystitis: Acute lower urinary tract infection. Vaginosis: Bacterial vaginosis. Vulvovaginitis: Candidiasis of vulva and vagina. Fully adjusted for sociodemographic factor (age, education level, income quartile, region of residency, and country of origin), parity, and other genital infections (Bartholin gland infection, cervicitis, pelvic-inflammatory-disease, salpingitis, urogenital herpes, and uteritis). ^a^ 2366 cases were excluded. ^b^ 793 cases were excluded. ^c^ 903 cases were excluded.

## Discussion

This nationwide study identifies associations between the common urogenital infections bacterial vaginosis and cystitis and cervical cancer *in situ* as well as cervical cancer. An earlier meta-analysis^24^ with up to 4644 included patients showed similar results with bacterial vaginosis associated with cervical carcinoma *in situ,* whereas our study included more than 62,000 cases*.* In addition, our results also added an examination of the associations between bacterial vaginosis and cystitis and cervical cancer to previous literature, which to our knowledge has not been shown before. The results remained after adjusting for sociodemographic factors, parity, and other genital infections (e.g., cervicitis, salpingitis, and urogenital herpes). This indicates that these common urogenital infections might be independently related to HPV linked neoplasia. Furthermore, an increased number of bacterial vaginosis events also showed a gradient association with cervical carcinoma *in situ,* and bacterial vaginosis remained significantly associated with both cervical cancer and cervical carcinoma *in situ* in the sensitivity model were the urogenital infection had to occur prior to the two outcomes – suggesting a potential causality behind this association.

Bacterial vaginosis as a co-factor in the natural history of HPV infection and related diseases remains unclear. However, this study presents new evidence suggesting this to be possible. One putative explanation behind the association might be the fact that bacterial vaginosis promotes the acquisition and persistence of HPV infection. Bacterial vaginosis is associated with profound changes in the microbiome and the physicochemical and immunological environment of the vagina^5^^,^^8^^,^^29^^,^^30^ and it has been suggested that such alterations may promote persistent HPV infections and thus the development of cervical neoplasia.^12^, ^13^, ^14^^,^^16^^,^^29^ For example, bacterial vaginosis associated bacteria and depletion of certain *Lactobacillus* spp., hydrogen peroxidase production and increased pH have been linked to bacterial vaginosis and HPV (partly to cystitis and vulvovaginitis too).^4^^,^^5^^,^^8^, ^9^, ^10^, ^11^, ^12^, ^13^, ^14^, ^15^, ^16^, ^17^ Furthermore, bacterial vaginosis is associated with epithelial cell damaging biochemical changes in vaginal secretions (including production of metabolites such as propionate and butyrate), anaerobic bacteria released volatile amines (inducing the characteristic fishy malodour),^29^ and alterations in inflammatory cytokine profile^30^ – which also may promote the development of cervical lesions and neoplasia. Moreover, women with bacterial vaginosis and cervical lesions have shown a similar increase in the local cervical immune profile of certain cytokines and nitric oxide.^31^ All this considered, the chronic cervical inflammation due to a combination of bacterial vaginosis and HPV infection seems like a putative explanation behind the gradient and significant associations between bacterial vaginosis and cervical neoplasia in the present study. Bacterial vaginosis may be an associated cofactor^22^ to the HPV infection inducing high-grade cervical lesions in persistent HPV-infected women. Therefore, persistent HPV infections in women with a certain vaginal microbiota prone to dysbiotic conditions may be more prone to acquire a more rapid dysplasia progression, or the cervical dysplasia induced by the combination of HPV infection and bacterial vaginosis. Further studies are needed to clarify the causal pathogenic pathways for better targeted treatments and interventions.

The associations between cystitis, vulvovaginitis, and cervical neoplasia were weaker than that of bacterial vaginosis. For vulvovaginitis, after adjusting for other urogenital infections and potential confounders as well as in the sensitivity analysis (exposure prior to outcome), the inverse association between this urogenital condition and cervical cancer remained similar to previous smaller studies.^24^ However, the association with cervical carcinoma *in situ* was somewhat inconsistent throughout the different models and the relationship diminished in the sensitivity analysis. Altogether, this could somewhat question any causal relationship, but further studies are needed to make a firmer conclusion. To our knowledge, the mechanism behind the possible inverse association between vulvovaginitis and cervical neoplasia has also not been fully understood. However, the vaginal microbiome or as previously suggested other immune enhancing properties^24^ might hold a possible explanation. The relationship between cystitis and cervical neoplasia has to our knowledge not either been studied before. We found this very common urogenital condition to be associated with cervical neoplasia and increased numbers of infections seemed to increase this risk. However, the sensitivity analysis failed to confirm this association. Altogether, this indicates that while cystitis and cervical neoplasia was found to be significantly associated, a causal relationship seems less plausible due to the lack of temporality. On the other hand, it is possible that cervical neoplasia might be a risk factor for cystitis, explaining the relatively strong association found in this present study. Some evidence suggests that the vaginal microbiome might have a role in this relationship,^1^^,^^25^ but the possibility of externally shared risk factors such as sexual behaviour remains.^10^^,^^14^ Future prospective studies are needed to confirm this novel finding and its potential causal association.

The most important limitations in this study were that we do not have access to microbiological data nor data on HPV-vaccination or cervical screening. HPV vaccinated young women have been shown to have less cervical cancer,^32^ which also counts for women attending past and ongoing cervical screening programs.^33^ Although we were unable to address these potential confounders directly, this is unlikely to have a large impact on the results. HPV vaccination was introduced to young Swedish women in 2006 and in the childhood vaccination program in 2012, leaving only part of the youngest age group the possibility to be affected by this potential confounder in this present cohort, which probably would not be differential. Besides, the four valent HPV-vaccine given to ten- to 12-year-old girls from 2012 does not cover all serotypes of high-risk HPV.^33^ As for cervical cancer screening, we assessed this potential differential confounder indirectly by adjusting for known sociodemographic risk factors, for non-attending in the Swedish cervical screening program.^27^ Moreover, although several of the urogenital infections assessed in this present study share sexual behaviour as a risk factor for cervical cancer,^1^, ^2^, ^3^^,^^5^^,^^20^^,^^25^ we were unable to directly consider sexual behaviour and other lifestyle factors due to the nationwide nature of our data. However, we had access to diagnosis of infections strongly related to sexually transmitted pathogens (e.g., cervicitis, uteritis, salpingitis, urogenital herpes simplex) and could thus adjust for these without attenuation of the findings. Marital status was also adjusted for in a sensitivity analysis without any large change of the findings. All this considered, this study was likely able to indirectly adjust for sexual behaviour. Furthermore, since sociodemographic factors, especially country of origin, have been found to be related to urogenital infections in Sweden,^6^ we adjusted for these factors and also examined for potential interactions between the three urogenital infections and country of origin in relation to cervical cancer, but no significant effect was found. Due to the design of the study, we could not take into account when in time a new urogenital diagnosis occurred in relation to the prior. Therefore, we could not either conclude if the increased risk of cervical neoplasia seen with increased number of infections was attributed to recurrent or new infections, or the same infection with repeated contacts. Finally, the risks of reverse causality bias and confounding by indication due to the design of the study should also be mentioned. Altogether, although the present study provides novel insight on potential risk factors for cervical cancer and cervical carcinoma *in situ* on a nationwide basis, future prospective studies including microbiological data are needed to confirm these findings and establish the direction of these relationships – especially regarding cystitis. However, we believe the limitations were balanced by the strengths of this study.

The most important strengths with this study are that it included data from nationwide registers, and the consistency between our results and previous findings strengthens that our data sources can be used to identify these urogenital infections. For example, the findings related to bacterial vaginosis and vulvovaginitis were similar to smaller studies of various designs (which include microbiological data),^23^^,^^24^ the incidence rate and the bimodal age distribution of cervical cancer were in-line with previous incidence studies in Scandinavia,^33^ and the standardised incidence rate of cervical cancer was as expected only slightly higher than the world age standardised incidence rate of cervical cancer for Sweden in 2018.^26^ The association between genital infections and cervical neoplasia were also in accordance with the probable co-factoring effect^21^^,^^22^ of such infections on HPV-originating cervical neoplasia. Furthermore, our study had access to hitherto quite unique nationwide primary healthcare data, used to identify medically attended urogenital conditions commonly diagnosed and managed in primary healthcare settings.^6^^,^^7^ Consequently, no other population-based studies of this size have been conducted on the relationship between these common urogenital infections and cervical neoplasia. All this considered, the main findings of this study can most likely be considered representative and important new information. Most specifically, the strong association (including in the sensitivity analyses), consistency with previous smaller studies and the plausible mechanisms based on previous research suggest a plausible causal relationship and biological gradient between bacterial vaginosis and HPV related neoplasia. The previously unstudied association between cystitis and cervical neoplasia warrants further research attention as this is one of the most common infections in women worldwide.^1^^,^^6^

The clinical implication is that common medically attended urogenital infective conditions such as cystitis and particularly bacterial vaginosis might now be recognised as independently associated with cervical cancer, the latter as a plausible risk factor. However, although this nationwide data provides statistical significance, the clinical significance for individual women is likely to have a more modest impact, especially for women suffering from vulvovaginitis and cystitis. Nevertheless, increased preventive measures in women suffering from bacterial vaginosis and specifically those suffering frequent episodes (and even frequent cystitis infections) could be considered, while at the same time observing if such interventions would have a positive health effect. For example, genital HPV-analyses^34^ and HPV vaccination^32^ could be offered women who seek medical attention for bacterial vaginosis or even suffer from frequent cystitis if they have not taken part of the cervical cancer screening program or are unvaccinated.

In conclusion, this large nationwide study suggests that certain very common urogenital infective conditions attended in primary healthcare might serve as important predictors in identifying women at increased risk of cervical cancer. Both bacterial vaginosis and cystitis seem to be independently associated with cervical neoplasia (but only vaginosis had a temporal association), while vulvovaginitis was inversely associated with this malignancy. The association between bacterial vaginosis, urinary cystitis, and cervical cancer has not been described before. A biological plausibility behind the association between bacterial vaginosis and cervical neoplasia might exist. More studies are needed, including on the causal mechanisms behind these associations, before generalized public health recommendations can be implemented based on these findings.

## Contributors

All authors have approved the final version of the manuscript. Concept: FJ, inspired by NFM. Development of idea and design: FJ, XL, and KS. Critical revision and approval of design: All authors. Access and acquisition of data: KS. Analysis and statistics: XL. Tables: XL and FJ. Interpretation of data: All authors. Literature search: FJ and CB. Drafting of manuscript: FJ with support from CB. Critical revision of the manuscript for intellectual content: KS, JDK, NFM, CB and XL. The authors attest that all listed authors meet the authorship criteria and that no others meeting the criteria have been omitted.

### Data availability statement

This study made use of several national registers and, owing to legal concerns, data cannot be made openly available. Further information regarding the health registries is available from the Swedish National Board of Health and Welfare: https:// www.socialstyrelsen.se/en/statistics-and-data/registers/. The code used in the analysis can be provided upon request.

## Declaration of interests

The authors have nothing to disclose.

## Appendix Supplementary materials

Image, application 1

Image, application 2

## References

[1] Hooton T.M., Scholes D., Hughes J.P. (1996). A prospective study of risk factors for symptomatic urinary tract infection in young women. N Engl J Med.

[2] Coudray M.S., Madhivanan P. (2020). Bacterial vaginosis-a brief synopsis of the literature. Eur J Obstet Gynecol Reprod Biol.

[3] Goncalves B., Ferreira C., Alves C.T., Henriques M., Azeredo J., Silva S. (2016). Vulvovaginal candidiasis: epidemiology, microbiology and risk factors. Crit Rev Microbiol.

[4] Kalia N., Singh J., Kaur M. (2020). Microbiota in vaginal health and pathogenesis of recurrent vulvovaginal infections: a critical review. Ann Clin Microbiol Antimicrob.

[5] Cherpes T.L., Hillier S.L., Meyn L.A., Busch J.L., Krohn M.A. (2008). A delicate balance: risk factors for acquisition of bacterial vaginosis include sexual activity, absence of hydrogen peroxide-producing lactobacilli, black race, and positive herpes simplex virus type 2 serology. Sex Transm Dis.

[6] Jansåker F., Li X., Sundquist K. (2021). Sociodemographic factors and uncomplicated cystitis in women aged 15–50 years: a nationwide Swedish cohort registry study (1997–2018). Lancet Reg Health Eur.

[7] Jansaker F., Frimodt-Moller N., Li X., Sundquist K. (2022). Novel risk factors associated with common vaginal infections: a nationwide primary health care cohort study: novel risk factors for vaginal infections. Int J Infect Dis.

[8] Ravel J., Gajer P., Abdo Z. (2011). Vaginal microbiome of reproductive-age women. Proc Natl Acad Sci U S A.

[9] Ceccarani C., Foschi C., Parolin C. (2019). Diversity of vaginal microbiome and metabolome during genital infections. Sci Rep.

[10] Gupta K., Stapleton A.E., Hooton T.M., Roberts P.L., Fennell C.L., Stamm W.E. (1998). Inverse association of H_2_O_2_-producing lactobacilli and vaginal Escherichia coli colonization in women with recurrent urinary tract infections. J Infect Dis.

[11] Donders G.G., Bosmans E., Dekeersmaecker A., Vereecken A., Van Bulck B., Spitz B. (2000). Pathogenesis of abnormal vaginal bacterial flora. Am J Obstet Gynecol.

[12] Norenhag J., Du J., Olovsson M., Verstraelen H., Engstrand L., Brusselaers N. (2020). The vaginal microbiota, human papillomavirus and cervical dysplasia: a systematic review and network meta-analysis. BJOG.

[13] Brotman R.M., Shardell M.D., Gajer P. (2014). Interplay between the temporal dynamics of the vaginal microbiota and human papillomavirus detection. J Infect Dis.

[14] Dareng E.O., Ma B., Adebamowo S.N. (2020). Vaginal microbiota diversity and paucity of lactobacillus species are associated with persistent hrHPV infection in HIV negative but not in HIV positive women. Sci Rep.

[15] Tortelli B.A., Lewis W.G., Allsworth J.E. (2020). Associations between the vaginal microbiome and candida colonization in women of reproductive age. Am J Obstet Gynecol.

[16] Cheng L., Norenhag J., Hu Y.O.O. (2020). Vaginal microbiota and human papillomavirus infection among young Swedish women. NPJ Biofilms Microbiomes.

[17] van de Wijgert J., Gill A.C., Chikandiwa A. (2020). Human papillomavirus infection and cervical dysplasia in HIV-positive women: potential role of the vaginal microbiota. AIDS.

[18] Parolin C., Marangoni A., Laghi L. (2015). Isolation of vaginal lactobacilli and characterization of anti-candida activity. PLoS One.

[19] Koshiol J., Lindsay L., Pimenta J.M., Poole C., Jenkins D., Smith J.S. (2008). Persistent human papillomavirus infection and cervical neoplasia: a systematic review and meta-analysis. Am J Epidemiol.

[20] International Collaboration of Epidemiological Studies of Cervical Cancer (2007). Comparison of risk factors for invasive squamous cell carcinoma and adenocarcinoma of the cervix: collaborative reanalysis of individual data on 8,097 women with squamous cell carcinoma and 1,374 women with adenocarcinoma from 12 epidemiological studies. Int J Cancer.

[21] Munoz N., Castellsague X., Berrington de Gonzalez A., Chapter G.L. (2006). 1: HPV in the etiology of human cancer. Vaccine.

[22] Castle P.E., Chapter G.A.R. (2003). 4: Genital tract infections, cervical inflammation, and antioxidant nutrients–assessing their roles as human papillomavirus cofactors. J Natl Cancer Inst Monogr.

[23] Gillet E., Meys J.F., Verstraelen H. (2012). Association between bacterial vaginosis and cervical intraepithelial neoplasia: systematic review and meta-analysis. PLoS One.

[24] Liang Y., Chen M., Qin L., Wan B., Wang H. (2019). A meta-analysis of the relationship between vaginal microecology, human papillomavirus infection and cervical intraepithelial neoplasia. Infect Agent Cancer.

[25] Plummer M., Peto J., Franceschi S., International Collaboration of Epidemiological Studies of Cervical Cancer (2012). Time since first sexual intercourse and the risk of cervical cancer. Int J Cancer.

[26] Arbyn M., Weiderpass E., Bruni L. (2020). Estimates of incidence and mortality of cervical cancer in 2018: a worldwide analysis. Lancet Glob Health.

[27] Broberg G., Wang J., Ostberg A.L. (2018). Socio-economic and demographic determinants affecting participation in the Swedish cervical screening program: a population-based case-control study. PLoS One.

[28] Commission E, Eurostat, PM, et al. Revision of the european standard population: report of Eurostat's task force: 2013 edition: publications office; 2013.

[29] Wolrath H., Forsum U., Larsson P.G., Boren H. (2001). Analysis of bacterial vaginosis-related amines in vaginal fluid by gas chromatography and mass spectrometry. J Clin Microbiol.

[30] Cauci S. (2004). Vaginal immunity in bacterial vaginosis. Curr Infect Dis Rep.

[31] Tavares-Murta B.M., de Resende A.D., Cunha F.Q., Murta E.F. (2008). Local profile of cytokines and nitric oxide in patients with bacterial vaginosis and cervical intraepithelial neoplasia. Eur J Obstet Gynecol Reprod Biol.

[32] Lei J., Ploner A., Elfstrom K.M. (2020). HPV vaccination and the risk of invasive cervical cancer. N Engl J Med.

[33] Sundqvist A. (2021).

[34] Asciutto K.C., Ernstson A., Forslund O., Borgfeldt C. (2018). Self-sampling with HPV mRNA analyses from vagina and urine compared with cervical samples. J Clin Virol.

