# Supplementary material for: The association between common urogenital infections and cervical neoplasia – A nationwide cohort study of over four million women (2002–2018)
Source: Lancet Reg Health Eur. 2022 Apr 21;17:100378. doi: 10.1016/j.lanepe.2022.100378 (PMC9039866; doi:10.1016/j.lanepe.2022.100378)
Supplement: Supplementary file 1 [file mmc1.docx]

**Capitation for supplementary material**

**Table S1-S5**

**Table S1.** Number of cases of cystitis, vulvovaginitis, and vaginosis in women diagnosed during the study period

**Table S2.** Sensitivity analysis on the fully adjusted association between three common urogenital infections (analysed together) and cervical cancer and carcinoma *in situ,* including marital status in the adjustments.

**Table S3.** The association between the number of common urogenital infections (analysed separately) and cervical cancer and cervical carcinoma *in situ*

**Table S4.** The association between the number of common urogenital infections (analysed separately) and cervical carcinoma *in situ*

**Table S5.** Characteristics of the study population (N = 4 120 557)

**Figure S1-2**

**Figure S1.** Kaplan-Meier survival estimates on time to cervical cancer in relation to common urogenital conditions

**Figure S2**. Kaplan-Meier survival estimates on time to cervical carcinoma *in situ* in relation to common urogenital conditions
