# Supplementary material for: The association between common urogenital infections and cervical neoplasia – A nationwide cohort study of over four million women (2002–2018)
Source: Lancet Reg Health Eur. 2022 Apr 21;17:100378. doi: 10.1016/j.lanepe.2022.100378 (PMC9039866; doi:10.1016/j.lanepe.2022.100378)

| **Table S1.** Number of cases of cystitis, vulvovaginitis, and vaginosis in women diagnosed during the study period | | | | |
| --- | --- | --- | --- | --- |
|  | **Cystitis** | **Vulvovaginitis** | **Vaginosis** | **All** |
| **Cystitis** | 1 194 561 | 82 358 | 57 906 | 1 194 561 |
| **Vulvovaginitis** | 82 358 | 167 864 | 24 551 | 167 864 |
| **Vaginosis** | 57 906 | 24 551 | 124 287 | 124 287 |
| **All** | 1 194 561 | 167 864 | 124 287 |  |
| Cystitis: Acute lower urinary tract infection. Vaginosis: Bacterial vaginosis. Vulvovaginitis: Candidiasis of vulva and vagina. | | | | |

| **Table S2.** Sensitivity analysis on the fully adjusted association between three common urogenital infections (analysed together) and cervical cancer and carcinoma *in situ,* including marital status in the adjustments. | | | | | | | | | | |
| --- | --- | --- | --- | --- | --- | --- | --- | --- | --- | --- |
|  | **Cervical cancer** | | | |  | **Cervical carcinoma *in situ*** | | | | |
| Covariates | HR* | 95% CI | | P-value |  | HR* | 95% CI | | P-value |  |
| **Cystitis** | 1∙23 | 1∙16 | 1∙29 | <∙0001 |  | 1∙10 | 1∙08 | 1∙12 | <∙0001 |  |
| (ref. non) |  |  |  |  |  |  |  |  |  |  |
| **Vaginosis** | 1∙27 | 1∙12 | 1∙43 | 0∙0002 |  | 1∙77 | 1∙73 | 1∙82 | <∙0001 |  |
| (ref. non) |  |  |  |  |  |  |  |  |  |  |
| **Vulvovaginitis** | 0∙80 | 0∙70 | 0∙91 | 0∙0010 |  | 1∙13 | 1∙10 | 1∙16 | <∙0001 |  |
| (ref. non) |  |  |  |  |  |  |  |  |  |  |
| HR: Hazards ratio. CI: Confidence interval. Cystitis: Acute lower urinary tract infection. Vaginosis: Bacterial vaginosis. Vulvovaginitis: Candidiasis of vulva and vagina. *Fully adjusted for sociodemographic factor (age, education level, income quartile, region of residency, and country of origin), parity, and other genital infections (Bartholin gland infection, cervicitis, pelvic-inflammatory-disease, salpingitis, urogenital herpes, and uteritis), as well as marital status (married/cohabiting or not). | | | | | | | | | | |

| **Table S3**. The association between the number of common urogenital infections (analysed separately) and cervical cancer and cervical carcinoma *in-situ* | | | | | | | | | | | | | | | | | | | | | | |
| --- | --- | --- | --- | --- | --- | --- | --- | --- | --- | --- | --- | --- | --- | --- | --- | --- | --- | --- | --- | --- | --- | --- |
|  | **Cystitis** | | | | | |  | **Vaginosis** | | | | | |  | | **Vulvovaginitis** | | | | | | |
| Covariates | No. | Person years | HR* | 95% CI | | P-value |  | No. | Person years | HR* | 95% CI | | P-value | |  | No. | Person years | HR* | 95% CI | | P-value |  |
| **No. of infections** (ref. No infection) | **Cervical cancer (5781 events)** | | | | | | | | | | | | | | | | | | | | | |
| One | 947 | 5 881 414 | 1∙18 | 1∙10 | 1∙27 | <∙0001 |  | 189 | 927 849 | 1∙33 | 1∙15 | 1∙54 | 0∙0001 | |  | 136 | 1 267 802 | 0∙73 | 0∙61 | 0∙87 | 0∙0003 |  |
| Two | 472 | 2 740 039 | 1∙27 | 1∙15 | 1∙40 | <∙0001 |  | 58 | 296 864 | 1∙26 | 0∙97 | 1∙63 | 0∙0852 | |  | 55 | 367 814 | 1∙01 | 0∙78 | 1∙32 | 0∙9167 |  |
| Three | 259 | 1 481 920 | 1∙29 | 1∙13 | 1∙46 | <∙0001 |  | 15 | 76 288 | 1∙23 | 0∙74 | 2∙05 | 0∙4179 | |  | 14 | 118 472 | 0∙80 | 0∙48 | 1∙36 | 0∙4133 |  |
| Four | 155 | 90 0178 | 1∙27 | 1∙08 | 1∙49 | 0∙0041 |  | 5 | 41 739 | 0∙75 | 0∙31 | 1∙80 | 0∙5137 | |  | 10 | 57 654 | 1∙19 | 0∙64 | 2∙22 | 0∙5792 |  |
| Five or more | 376 | 2 244 882 | 1∙22 | 1∙10 | 1∙36 | 0∙0003 |  | 10 | 40 677 | 1∙50 | 0∙81 | 2∙79 | 0∙2026 | |  | 6 | 70 304 | 0∙57 | 0∙26 | 1∙28 | 0∙1726 |  |
|  |  | *Linear trend P-value = 0∙1643* | | | | |  |  | *Linear trend P-value = 0∙7418* | | | | | |  |  |  | *Linear trend P-value = 0∙6212* | | | |  |
| **Other genital infections** | 429 | 1 587 309 | 1∙89 | 1∙71 | 2∙09 | <∙0001 |  | 429 | 1 587 309 | 1∙90 | 1∙71 | 2∙10 | <∙0001 | |  | 429 | 1 587 309 | 1∙97 | 1∙78 | 2∙18 | <∙0001 |  |
|  |  |  |  |  |  |  |  |  |  |  |  |  |  | |  |  |  |  |  |  |  |  |
| **No. of infections** (ref. Non) | **Cervical carcinoma in-situ (62 249 events)** | | | | | | | | | | | | | | | | | | | | | |
| One | 10 627 | 5 881 414 | 1∙10 | 1∙07 | 1∙12 | <∙0001 |  | 4086 | 927 849 | 1∙73 | 1∙68 | 1∙79 | <∙0001 | |  | 3212 | 1 267 802 | 1∙06 | 1∙02 | 1∙10 | 0∙0020 |  |
| Two | 4976 | 2 740 039 | 1∙11 | 1∙08 | 1∙15 | <∙0001 |  | 1417 | 296 864 | 1∙84 | 1∙75 | 1∙94 | <∙0001 | |  | 1145 | 367 814 | 1∙24 | 1∙17 | 1∙31 | <∙0001 |  |
| Three | 2598 | 1 481 920 | 1∙11 | 1∙07 | 1∙16 | <∙0001 |  | 416 | 76 288 | 1∙96 | 1∙78 | 2∙16 | <∙0001 | |  | 384 | 118 472 | 1∙25 | 1∙13 | 1∙38 | <∙0001 |  |
| Four | 1520 | 90 0178 | 1∙12 | 1∙06 | 1∙18 | <∙0001 |  | 230 | 41 739 | 2∙04 | 1∙79 | 2∙32 | <∙0001 | |  | 185 | 57 654 | 1∙27 | 1∙10 | 1∙46 | 0∙0014 |  |
| Five or more | 3057 | 2 244 882 | 1∙09 | 1∙05 | 1∙13 | <∙0001 |  | 263 | 40 677 | 2∙44 | 2∙16 | 2∙75 | <∙0001 | |  | 268 | 70 304 | 1∙44 | 1∙27 | 1∙62 | <∙0001 |  |
|  |  | *Linear trend P-value <∙001* | | | | |  |  | *Linear trend P-value <∙001* | | | | | |  |  |  | *Linear trend P-value = 0∙7235* | | | |  |
| **Other genital infections** | 6359 | 1 587 309 | 1∙62 | 1∙58 | 1∙67 | <∙0001 |  | 6359 | 1 587 309 | 1∙51 | 1∙47 | 1∙56 | <∙0001 | |  | 6359 | 1 587 309 | 1∙63 | 1∙58 | 1∙67 | <∙0001 |  |
| HR: Hazards ratio. CI: Confidence interval. No.: number. Cystitis: Acute lower urinary tract infection. Vaginosis: Bacterial vaginosis. Vulvovaginitis: Candidiasis of vulva and vagina. Other genital infections: Bartholin gland infection, cervicitis, pelvic-inflammatory-disease, salpingitis, urogenital herpes, and uteritis. *Fully adjusted for sociodemographic factors (age, education level, income quartile, region of residency and country of origin) other genital infections, and parity. Time period: 2002-2018. | | | | | | | | | | | | | | | | | | | | | | |

| **Table S4.** The association between the number of common urogenital infections (analysed separately) and cervical carcinoma *in situ* | | | | | | | | | | | | | | | | | | | | | | | | | | |
| --- | --- | --- | --- | --- | --- | --- | --- | --- | --- | --- | --- | --- | --- | --- | --- | --- | --- | --- | --- | --- | --- | --- | --- | --- | --- | --- |
|  |  | **Cystitis** | | | | | | |  | | **Vaginosis** | | | | | | |  | | **Vulvovaginitis** | | | | | |  |
|  | Covariates | No. | | HR* | 95% CI | | P-value | |  | | No. | | HR* | | 95% CI | | P-value |  | No. | | HR* | 95% CI | | P-value | | |
| **Number of infections** (ref. Non) | |  |  |  |  | |  | |  | |  | |  |  |  |  |  |  | |  |  |  |  | | |  |
| One to five | 20 682 | | 1∙12 | 1∙09 | 1∙16 | | <∙0001 | |  | | 6253 | | 1∙79 | 1∙74 | 1∙84 | <∙0001 |  | 5032 | | 1∙11 | 1∙09 | 1∙12 | <∙0001 | | |  |
| Six to ten | 1644 | | 1∙41 | 1∙20 | 1∙67 | | <∙0001 | |  | | 140 | | 2∙48 | 2∙10 | 2∙93 | <∙0001 |  | 136 | | 1∙09 | 1∙03 | 1∙14 | 0∙0010 | | |  |
| Above ten | 452 | | 1∙35 | 0∙92 | 1∙98 | | 0∙1291 | |  | | 19 | | 2∙26 | 1∙44 | 3∙54 | 0∙0004 |  | 26 | | 0∙99 | 0∙91 | 1∙09 | 0∙8863 | | |  |
| HR: Hazards ratio. CI: Confidence interval. No.: Number of events. Cystitis: Acute lower urinary tract infection. Vaginosis: Bacterial vaginosis. Vulvovaginitis: Candidiasis of vulva and vagina. Other genital infections: Bartholin gland infection, cervicitis, pelvic-inflammatory-disease, salpingitis, urogenital herpes, and uteritis. *Fully adjusted for sociodemographic factors (age, education level, income quartile, region of residency and country of origin) other genital infections, and parity. Time period: 2002-2018. | | | | | | | | | | | | | | | | | | | | | | | |  |  |  |

| **Supplementary Table S5.** Characteristics of the study population (N = 4 120 557) | | |
| --- | --- | --- |
|  | No. | % |
| **Urogenital condition** |  |  |
| Cystitis | 1 194 561 | 29.0 |
| Vulvovaginitis | 167 864 | 4.1 |
| Bacterial vaginosis | 124 287 | 3.0 |
| **Age at baseline (years)** |  |  |
| 15-24 | 674 120 | 16.4 |
| 25-34 | 677 650 | 16.4 |
| 35-44 | 635 122 | 15.4 |
| 45-64 | 1 174 811 | 28.5 |
| ≥ 65 | 958 854 | 23.3 |
| **Educational level (years)** |  |  |
| ≤ 12 | 2 718 970 | 66.0 |
| > 12 | 1 401 587 | 34.0 |
| **Family income** |  |  |
| Low | 1 029 399 | 25.0 |
| Middle | 2 060 806 | 50.0 |
| High | 1 030 352 | 25.0 |
| **Region of residence** |  |  |
| Large cities | 2 270 266 | 55.1 |
| Southern Sweden | 1 271 384 | 30.9 |
| Northern Sweden | 578 907 | 14.0 |
| **Marital status** |  |  |
| Married/cohabiting | 1 721 323 | 41.8 |
| Unmarried/Divorced/Widowed | 2 399 234 | 58.2 |
| **Country of origin** |  |  |
| Sweden (country of birth) | 3 282 971 | 79.7 |
| Eastern Europe | 194 473 | 4.7 |
| Western countries | 242 373 | 5.9 |
| Middle East/North Africa | 191 713 | 4.7 |
| Africa (excluding North Africa) | 61 026 | 1.5 |
| Asia (excluding Middle East) and Oceania | 108 887 | 2.6 |
| Latin America and the Caribbean | 39 114 | 0.9 |
| **Parity** |  |  |
| Non | 2 227 780 | 54.1 |
| Yes | 1 892 777 | 45.9 |
| **Other genital infections** |  |  |
| Non | 3 975 389 | 96.5 |
| Yes | 145 168 | 3.5 |
| No.: number. Cystitis: Acute lower urinary tract infection. Vaginosis: Bacterial vaginosis.  Vulvovaginitis: Candidiasis of vulva and vagina. Other genital infections: Bartholin gland infection,  cervicitis, pelvic-inflammatory-disease, salpingitis, urogenital herpes, and uteritis | | |

**Figure S1.** Kaplan-Meier survival estimates on time to cervical cancer in relation to three common urogenital conditions


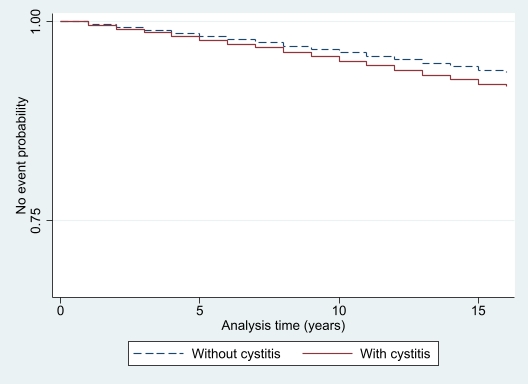

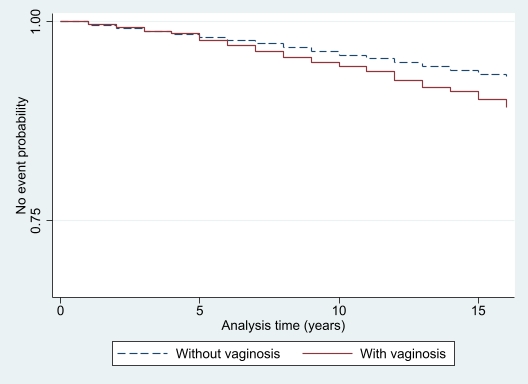

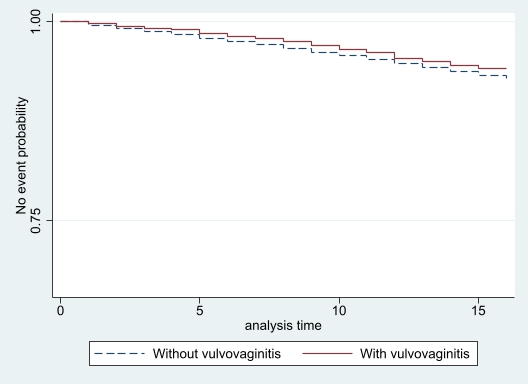


**Figure S2**. Kaplan-Meier survival estimates on time to cervical carcinoma *in situ* in relation to three common urogenital conditions


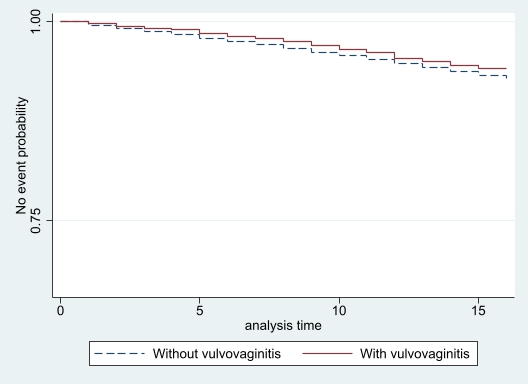

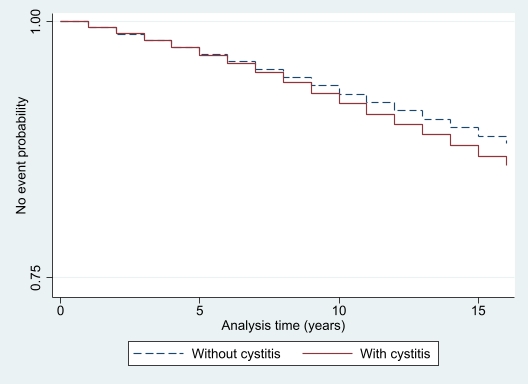

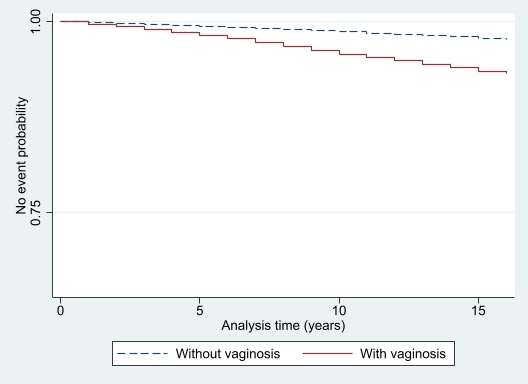

Supplement: Supplementary file 2 [file mmc2.docx]
